# *Bacteroides uniformis* CECT 7771 alleviates inflammation within the gut-adipose tissue axis involving TLR5 signaling in obese mice

**DOI:** 10.1038/s41598-021-90888-y

**Published:** 2021-06-03

**Authors:** Emanuel Fabersani, Kevin Portune, Isabel Campillo, Inmaculada López-Almela, Sergio Montserrat-de la Paz, Marina Romaní-Pérez, Alfonso Benítez-Páez, Yolanda Sanz

**Affiliations:** Microbial Ecology, Nutrition and Health Research Unit, Institute of Agrochemistry and Food Technology, National Research Council (IATA-CSIC), C/ Catedrático Agustín Escardino Benlloch 7, 46980 Paterna, Valencia Spain

**Keywords:** Immunology, Microbiology, Physiology

## Abstract

This study investigated the immune mechanisms whereby administration of *Bacteroides uniformis* CECT 7771 reduces metabolic dysfunction in obesity. C57BL/6 adult male mice were fed a standard diet or a Western diet high in fat and fructose, supplemented or not with *B. uniformis* CECT 7771 for 14 weeks. *B. uniformis* CECT 7771 reduced body weight gain, plasma cholesterol, triglyceride, glucose, and leptin levels; and improved oral glucose tolerance in obese mice. Moreover, *B. uniformis* CECT 7771 modulated the gut microbiota and immune alterations associated with obesity, increasing Tregs and reducing B cells, total macrophages and the M1/M2 ratio in both the gut and epididymal adipose tissue (EAT) of obese mice. *B. uniformis* CECT 7771 also increased the concentration of the anti-inflammatory cytokine IL-10 in the gut, EAT and peripheral blood, and protective cytokines TSLP and IL-33, involved in Treg induction and type 2 innate lymphoid cells activation, in the EAT. It also restored the obesity–reduced TLR5 expression in the ileum and EAT. The findings indicate that the administration of a human intestinal bacterium with immunoregulatory properties on the intestinal mucosa helps reverse the immuno-metabolic dysfunction caused by a Western diet acting over the gut-adipose tissue axis.

## Introduction

Obesity has become a major global health challenge due to its increasing prevalence. In 2016, more than 1.9 billion adults (39%) 18 years and older were overweight and of these over 650 million (13%) were obese, according to the WHO^1^. Obesity frequently results in a state of chronic low-grade inflammation that is considered a precipitating factor of metabolic complications, such as type 2 diabetes, cardiovascular disease and non-alcoholic fatty liver disease^2^. Inflammation of the white adipose tissue (WAT) is considered a major driver of metabolic alterations and, therefore, has been investigated in depth. WAT inflammation is mediated by an overall increase in macrophages largely due to the recruitment of M1 (or classically activated) macrophages and reduction of anti-inflammatory M2 macrophages (or alternatively activated macrophages). This leads to overproduction of pro-inflammatory cytokines (e.g. IL-1β, IL-6, and TNF-α) in relation to anti-inflammatory (IL-4 and IL-10) ones^3^. Although macrophages are considered the ultimate effector cells producing cytokines which cause metabolic dysfunction, IFN-γ–secreting Th1 cells, CD8 + T cells, and B cells are also increased in the WAT and contribute to macrophage recruitment and immune activation in this tissue^4^. The WAT has been considered the main contributor to inflammation and metabolic dysfunction during obesity, but now it is known that this phenomenon affects multiple organs, including the brain, muscle, liver and gut^5^. The most recent evidence specifically supports that the intestinal immune system and the microbes that expand under exposure to unhealthy diets are additional drivers of inflammation in obesity^6–8^ and that this metabolic inflammation (“metainflammation”) can be initiated in the gut^9^.

The intestinal microbiota influences multiple aspects of immunity, both locally and systemically, allowing for the induction of pro-inflammatory or regulatory immune pathways that set the inflammatory tone of different tissues^10^. In experimental study models, gut microbiota alterations resulting from unhealthy diets have been causally related to immune and metabolic alterations associated with obesity presumably due to dysfunctions in the cross-talk between the gut and other peripheral organs, such as the liver and the adipose tissue^11,12^. Specific mechanisms whereby interactions between unhealthy diets and the gut microbiota contribute to metabolic inflammation include reduction in host intestinal antimicrobial peptide production, over-activation of innate immunity leading to pro-inflammatory cytokine production, and disruption of the gut barrier facilitating translocation of microbial products (e.g. LPS)^8,11,13^. In light of these findings, strategies to restore the functions of the gut microbiota to help recover the control over the immune-metabolic axis in obesity are being investigated, including the administration of prebiotic fibers or specific bacterial strains^11,14^.

Controversial evidence regarding the role of the gut microbiota’s two dominant phyla, Bacteroidetes and Firmicutes, in diet-induced obesity has been documented. Numerous observational and intervention studies have correlated a lean phenotype or weight loss to increases in the phylum Bacteroidetes (including the genera *Bacteroides* and *Prevotella*), although a number of studies have, however, established inverse associations between obesity and these bacterial taxa^15,16^. Observational studies also associated increased abundances of Bacteroidetes or *Bacteroides* spp. with Western diets (high in animal fat and protein) related to obesity^17^. Nonetheless, a recent study indicates that associations established so far between the increased abundance of *Bacteroides* and consumption of Western diets rich in animal fat/proteins were oversimplifications and that sub-genus diversity also matters^18^. Different components of the genus *Bacteroides* were, in fact, associated with either plant-based or animal-based diets, the latest usually related to obesity^18^. In fact, *Bacteroides* spp. are known to be equipped with a metabolic machinery specialized in the utilization of oligo- and polysaccharides derived from plants that are part of healthy diets^19^ and lead to the production of short-chain fatty acids^20^, which may have beneficial effects on glucose metabolism and satiety. Moreover, strains of *Bacteroides fragilis* show immunomodulatory properties, optimizing the systemic Th1/Th2 balance, and inducing Treg cell differentiation, reducing autoimmune disorders in experimental models^21–23^. In a previous study carried out by our research group, *B. uniformis* CECT 7771 demonstrated an ability to reduce body weight gain and liver steatosis in mice fed a high-fat diet (HFD)^24^. Nevertheless, the possible role of *B. uniformis* CECT 7771 in the regulation of the inflammatory tone associated with obesity remains to be investigated.

This study aimed to progress in the understanding of the cellular and molecular mechanisms mediating the beneficial effects of *B. uniformis* CECT 7771 in the metabolic phenotype of diet-induced obese mice. To this end, we have specifically investigated the effects of the oral administration of this bacterial strain on immune cell populations and inflammatory mediators that may contribute to metabolic inflammation during obesity in the gut, peripheral blood and the adipose tissue. The possible molecular mechanisms mediating the effects related to TLR signaling and the microbiota configuration have also been investigated in depth.

## Results

### *Bacteroides uniformis* CECT 7771 improves the metabolic phenotype of obese mice

**Figure 1:**
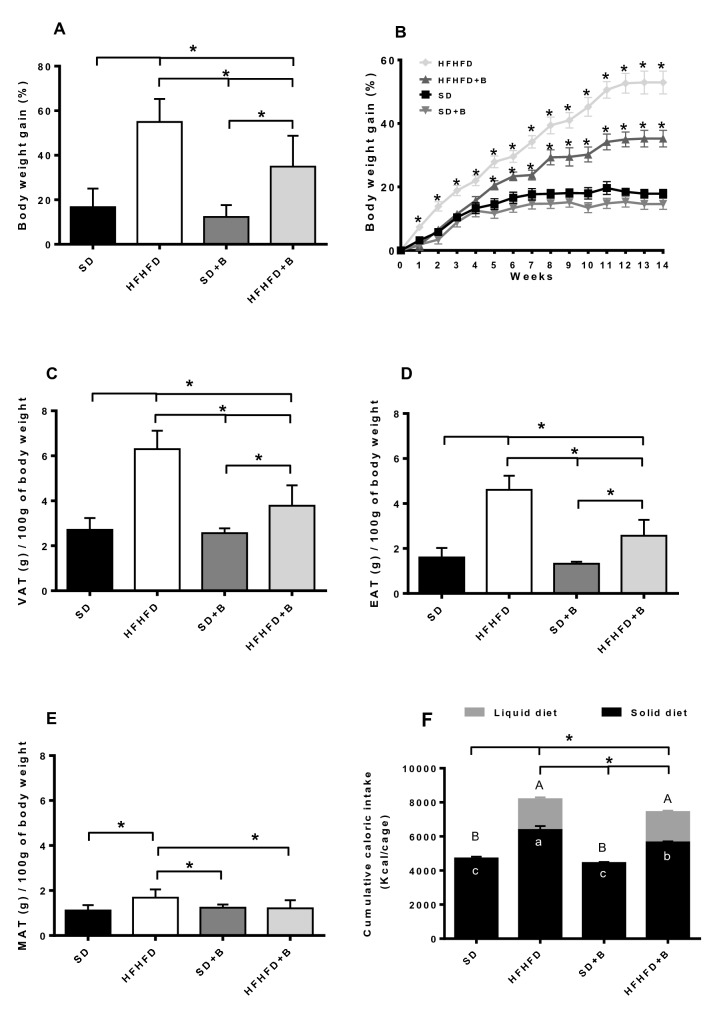
Anthropometric parameters and dietary intake. (**A**) Total body weight gain, (**B**) Weekly body weight gain  (**C**) Visceral adipose tissue (VAT) weight, (**D**) Epididymal adipose tissue (EAT) weight, (**E**) Mesenteric adipose tissue (MAT) weight, (**F**) Cumulative caloric intake from liquid diet, solid food and total caloric intake. Data are expressed as mean and standard error (vertical bars). Significant differences for liquid diets, solid foods, and total caloric intake are represented by uppercase letters, lowercase letters and stars, respectively. Statistically significant differences were established by ANOVA and post hoc student t test (*p* < 0.05).

The oral administration of *B. uniformis* CECT 7771 significantly reduced body weight gain approximately by 20% (*p* < 0.001) at the end of the intervention in the HFHFD-fed mice, but did not modify body weight gain in the SD-fed mice (Fig. 1A). Consistent with these findings, visceral adipose tissue (VAT), epididymal adipose tissue (EAT) and mesenteric adipose tissue (MAT) weights were significantly lower in obese mice fed *B. uniformis* (HFHFD + B group) (40%; *p* < 0.001, 44%, *p* < 0.001, and 28%, *p* = 0.005, respectively) than in obese mice fed placebo (HFHFD group) (Fig. 1C–E). Moreover, although the total caloric intake from both the solid and liquid parts of the diet was significantly higher in both mouse groups fed the HFHFD (*p* < 0.001) than in those fed the SD (Fig. 1F), the administration of *B. uniformis* CECT 7771 significantly reduced the total caloric intake, approximately by 11% in obese mice (HFHFD versus HFHFD + B *p* = 0.019), mainly by decreasing the caloric intake from solid food (Fig. 1F).

**Figure 2:**
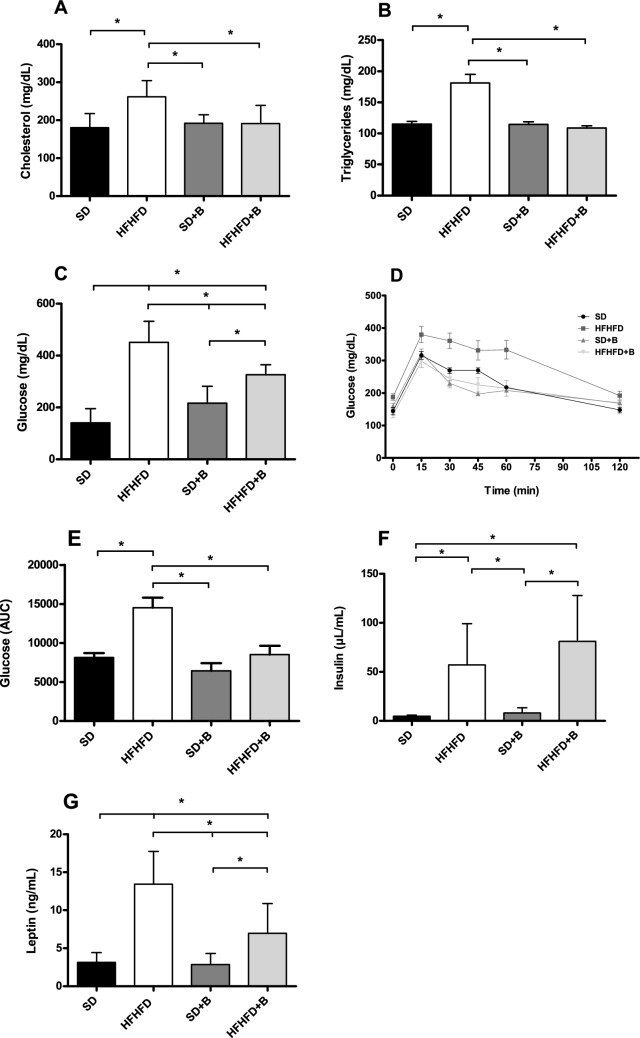
Plasma concentrations of (**A**) cholesterol, (**B**) triglycerides, (**C**) glucose, (**D**) glucose levels for individual time points in a glucose tolerance, (**E**) AUC values for glucose in a GTT, (**F**) insulin and (**G**) leptin. Data are expressed as mean and standard error (vertical bars). Statistically significant differences were established by ANOVA and post hoc student t test (*p* < 0.05).

The plasma concentrations of cholesterol, triglycerides, glucose, insulin, and leptin are shown in Fig. 2A–G. As expected, no changes in plasma values were observed between mice fed the SD and those fed the SD supplemented with *B. uniformis* CECT 7771. Compared to the SD group, the HFHFD group showed markedly increased (*p* < 0.001) plasma cholesterol (Fig. 2A), triglycerides (Fig. 2B) and glucose concentrations (Fig. 2C). Mice subjected to a glucose tolerance test displayed increased glucose levels in the HFHFD group compared to the other treatments for individual time points between 15 to 60 min, as well as overall AUC values (Fig. 2D,E). Insulin and leptin concentrations were also significantly elevated (*p* < 0.001) in the HFHFD group compared to the SD group (Fig. 2F,G). The administration of *B. uniformis* CECT 7771 to HFHF-fed mice (HFHFD + B group) significantly reduced plasma cholesterol (26%, *p* = 0.043), triglycerides (40%, *p* < 0.001), glucose (27%, *p* = 0.026), and leptin (48%, *p* = 0.019) concentrations compared to obese mice fed placebo (HFHFD group) (Fig. 2A–E, G).

### *Bacteroides uniformis* CECT 7771 restores the adaptive and innate immune cell imbalances of obese mice

The effects of *B. uniformis* CECT 7771 administration on lymphocyte and macrophage populations in peripheral blood, intestinal Peyer’s Patches (PP), and EAT from different mouse groups are shown in Table 1. Compared to the SD group, the HFHFD group showed increased proportions of B cells and reduced Tregs in peripheral blood, intestinal PP and EAT (*p* =  < 0.001–0.017). The administration of *B. uniformis* in obese mice fed a HFHFD effectively reduced the proportion of B cells (*p* =  < 0.001–0.006) and increased T regs (*p* = 0.013–0.041) in all tested compartments of obese mice (HFHFD + B versus HFHFD). Obese mice fed a HFHFD also showed increased proportions of total macrophages and of the M1/M2 ratio in PP and EAT compared to the SD group (*p* < 0.001–0.020), but these HFHFD-induced alterations were significantly reduced by the administration of *B. uniformis* CECT 7771 in both tissues (HFHFD + B versus HFHFD, *p* < 0.001–0.019).

**Table 1:** Effects of *Bacteroides uniformis* CECT 7771 on adaptive and innate immunity from mice in all experimental groups.

Cell population	Experimental groups											
	SD		HFHFD		SD + B		HFHFD + B		*p* value			
	Mean	*se*	Mean	*se*	Mean	*se*	Mean	*se*	HFHFD *vs* SD	SD + B *vs* SD	HFHFD + B *vs* HFHFD	HFHFD + B *vs* SD
**Peripheral blood**												
B cells (%)	22.19	3.13	60.18	10.58	28.92	6.96	18.85	5.30	0.011*	0.904	0.006*	0.986
Regulatory T cells (%)	5.69	0.73	0.75	0.20	6.75	1.76	4.99	0.40	0.017*	0.868	0.041*	0.955
**Peyer’s patches**												
B cells (%)	2.98	0.59	6.16	0.52	2.77	0.40	3.49	0.15	0.001*	0.987	0.006*	0.847
Regulatory T cells (%)	20.50	4.09	4.00	0.57	15.75	2.13	20.50	3.20	0.013*	0.711	0.013*	0.999
Total macrophages (%)	6.15	1.17	18.37	2.80	8.23	2.01	7.73	0.70	0.002*	0.856	0.007*	0.929
M1/M2 macrophage ratio	1.09	0.42	6.29	1. 99	1.88	0.41	1.07	0.27	0.020*	0.950	0.019*	0.059
**Epididymal adipose tissue**												
B cells (%)	1.21	0.22	5.30	0.20	0.66	0.10	2.65	0.10	< 0.001*	0.145	< 0.001*	< 0.001*
Regulatory T cells (%)	13.11	0.87	4.18	0.40	10.60	2.35	9.68	0.76	0.001*	0.832	0.038*	0.291
Total macrophages (%)	10.91	2.36	61.84	9.203	12.54	3.00	19.68	3.11	< 0.001*	0.996	< 0.001*	0.645
M1/M2 macrophage ratio	2.45	0.68	8.80	0.36	2.19	0.19	5.03	0.89	< 0.001*	0.989	0.003*	0.043*

SD group: control mice received a standard diet (SD) plus placebo; HFHFD group: obese mice received a high-fat high-fructose diet (HFHFD) plus placebo; SD + B group: control mice received a SD and a daily dose of 1 × 10^8^ CFU *Bacteroides uniformis* CECT 7771; HFHFD + B group: obese mice received HFHFD and a daily dose of 1 × 10^8^ CFU *Bacteroides uniformis* CECT 7771 by gavage during 14 weeks. Data are expressed as mean and standard error (*se*) of each mouse group (n = 10 per group). *Significant differences were established by ANOVA and post hoc student *t* test (*p* < 0.050).

### *Bacteroides uniformis* CECT 7771 regulates the cytokine network driving obesity-associated inflammation in mice

The effects of *B. uniformis* CECT 7771 administration on cytokine concentrations in the peripheral blood, EAT and ileum (PP) from control and obese mice are shown in Table 2. The direct inflammatory effects of the HFHFD in peripheral tissues (EAT) were reflected in the reduction of the anti-inflammatory and protective cytokines IL-10, IL-33 and TSLP compared to SD mice (*p* = 0.009–0.022). These cytokine alterations were reversed by the administration of *B. uniformis* CECT 7771 in obese mice (HFHFD + B versus HFHFD, *p* = 0.011–0.048). The role of *B. uniformis* CECT 7771 in the promotion of intestinal immune homeostasis was also translated into systemic effects in peripheral blood and locally in the gut (Peyer's patches) of obese mice. The administration of *B. uniformis* CECT 7771 ameliorated the HFHFD-induced alterations in peripheral blood concentrations of pro-inflammatory cytokines (IL-1α, and TNF-α) and the anti-inflammatory cytokine IL-10, and also increased IL-5 (*p* = 0.015–0.028), overall reducing the inflammatory tone. In PP, the HFHFD increased the concentration of IFNγ compared to the SD (*p* = 0.012), but *B. uniformis* CECT 7771 administration reversed this effect on IFNγ (*p* = 0.002) and also increased the concentrations of IL-10 (*p* = 0.032) in obese mice.

**Table 2:** Effects of *Bacteroides uniformis* CECT 7771 on cytokine concentrations in plasma and tissues from mice in all experimental groups.

Cell population	SD		HFHFD		SD + B		HFHFD + B		*p* value			
	Mean	*se*	Mean	*se*	Mean	*se*	Mean	*se*	HFHFD *vs* SD	SD + B *vs* SD	HFHFD + B *vs* HFHFD	HFHFD + B *vs* SD
**Peripheral blood**												
IL-1α (pg/mL)	1067.96	167.88	2591.65	740.56	229.93	86.30	710.28	223.42	0.047*	0.466	0.019*	0.919
IL-5 (pg/mL)	75.34	30.97	30.54	30.54	316.07	82.82	312.36	67.19	0.945	0.041*	0.015*	0.045*
IL-10 (pg/mL)	51.18	20.84	3.00	3.00	70.62	18.17	87.29	25.59	0.038*	0.884	0.028*	0.545
IL-13 (pg/mL)	2122.85	483.78	276.66	202.83	1121.60	391.20	552.60	310.00	0.011*	0.245	0.948	0.033*
TNF-α (pg/mL)	87.26	13.36	2028.2	806.0	207.80	169.80	250.00	153.90	0.022*	0.996	0.038*	0.992
**Peyer’s patches**												
IL-10 (pg/ g )	3846.83	683.99	2273.64	194.51	3090.06	266.77	4337.10	604.40	0.133	0.692	0.032*	0.890
IFNγ (pg/ g )	861.46	617.66	4142.19	598.51	627.70	378.15	1097.15	430.35	0.012*	0.988	0.002*	0.988
IL-33 (pg/ g)	24.10	1.10	29.73	2.55	23.92	0.53	25.82	2.11	0.142	0.999	0.420	0.899
TSLP (pg/ g )	10.76	1.67	16.80	4.67	10.75	1.89	13.52	2.72	0.504	0.999	0.862	0.912
**Epididymal adipose tissue**												
IL-10 (pg/ g )	52,878.71	9812.70	11,113.52	1555.77	57,114.54	8570.28	41,770.15	12,392.59	0.018*	0.987	0.038*	0.819
IFNγ (pg/ g )	14,383.86	5899.76	23,018.19	5164.28	8224.68	2990.85	10,334.88	5137.00	0.609	0.812	0.292	0.936
IL-33 (pg/ g)	8365.26	1295.68	2639.40	1234.19	4995.60	1344.29	8943.69	1228.05	0.022*	0.273	0.011*	0.988
TSLP (pg/ g)	4149.40	947.55	832.28	244.10	6917.25	842.20	3024.91	252.82	0.009*	0.034*	0.048*	0.628

SD group: control mice received a standard diet (SD) plus placebo; HFHFD group: obese mice received a high-fat high-fructose diet (HFHFD) plus placebo; SD + B group: control mice received a SD and a daily dose of 1 × 10^8^ CFU *Bacteroides uniformis* CECT 7771; HFHFD + B group: obese mice received HFHFD and a daily dose of 1 × 10^8^ CFU *Bacteroides uniformis* CECT 7771 by gavage during 14 weeks. Data are expressed as mean and standard error (*se*) of each mouse group (n = 10 per group). *Significant differences were established by ANOVA and post hoc student *t* test (*p* < 0.050).

### Induced changes in TLRs by diet and *B. uniformis* CECT 7771

**Figure 3:**
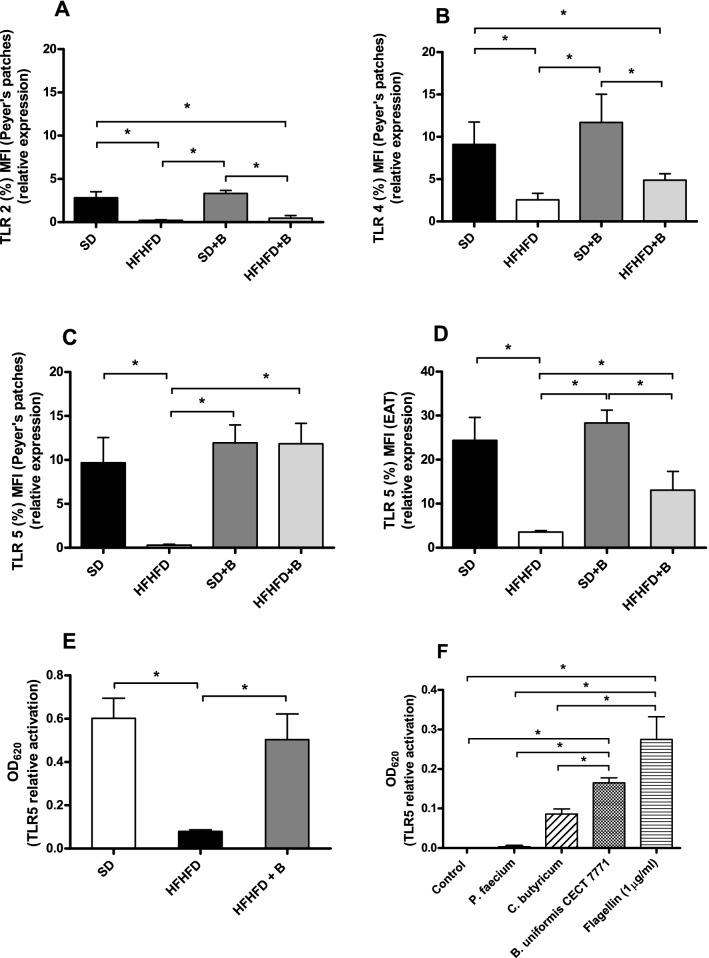
Relative expression of (**A**) TLR2, (**B**) TLR4 and (**C**) TLR5 from Peyer´s patches and (**D**) TLR5 from epididymal adipose tissue. Relative activation of TLR5 using HEK-Blue hTLR5 cells with (**E**) fecal samples from the SD, HFHFD and HFHFD + B experimental groups and (**F**) individual bacterial cultures of *Phascolarctobacterium faecium* (negative control), *Clostridium butyricum* (positive control), *B. uniformis* CECT 7771, and recombinant flagellin (RecFLA-ST, 1 μg/ml) (positive control). Control samples consisting of endotoxin-free water were included as negative controls. Data are expressed as mean and standard error (vertical bars). Statistically significant differences were established by ANOVA and post hoc student t test (*p* < 0.05).

To understand the molecular pathways mediating the effects of *B. uniformis* CECT 7771 in obesity, we analyzed the expression of TLRs in intestinal PP and EAT. The results show that in intestinal PP the HFHFD down-regulated the TLR2, TLR4, and TLR5 protein expression compared to SD (Fig. 3A, C), whereas *B. uniformis* CECT 7771 normalized the expression of TLR5 (Fig. 3C, *p* < 0.001) in obese mice levels compared to SD mice. Similar effects of HFHFD and *B. uniformis* CECT 7771 on TLR5 expression were detected in EAT (Fig. 3D), but with a lower magnitude than in the PP. In order to identify the possible in vivo activators of TLR5, in vitro experiments were conducted using HEK-Blue™ hTLR5 cells stimulated with fecal samples from the different experimental mouse groups and pure cultures of *B. uniformis* CECT 7771 (Fig. 3E,F). The results using HEK-Blue™ hTLR5 cells confirmed that TLR5 relative activation was significantly reduced when using fecal samples from the HFHFD-fed mice as stimulus and restored when using fecal samples from obese mice fed with *B. uniformis* CECT 7771 (Fig. 3E). Furthermore, pure cultures of *B. uniformis* CECT 7771 also significantly activated TLR5 and even more than a positive control strain of *C. butyricum* at similar cell concentrations, while bacterial cultures used as a negative control (*P. faecium*) did not activate TLR5 (Fig. 3F).

### Gut microbiota-induced changes by the diet and *B. uniformis* CECT 7771

**Figure 4:**
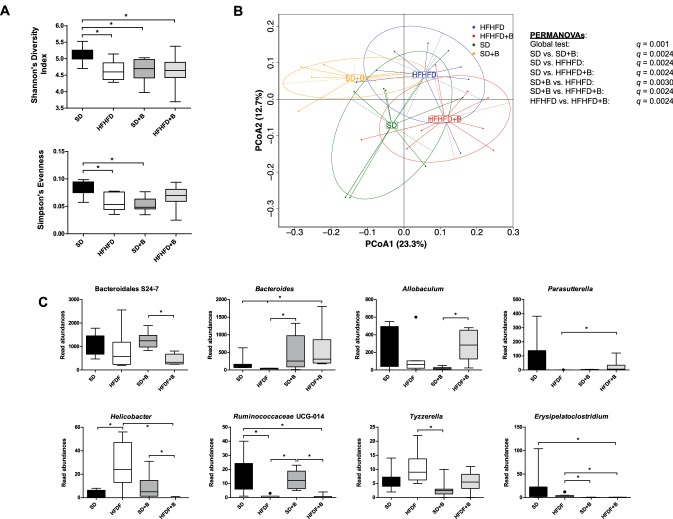
(**A**) Alpha diversity indices (Shannon’s diversity index, Simpson’s index, Simpson’s reciprocal index and Simpson’s evenness) from each treatment group. (**B**) Principle coordinates analysis (PCoA) plot using generalized UniFrac distances comparing microbial communities from each treatment group. Group means are indicated by the center of each ellipse. Distance-based non-parametric PERMANOVA tests were conducted at a global level as well as pairwise comparisons of treatment groups. *p* values of all pairwise comparisons were corrected for multiple comparisons (q) using false discovery rate. (**C**) Boxplots of gut microbiota taxonomic groups that demonstrated significant differences between treatment groups. Significant differences (*p* < 0.05) between treatment groups are represented with *.

Alpha diversity (Simpson’s diversity index) was significantly reduced (*p* < 0.05) in all treated mouse groups (HFHFD, SD + B, and HFHFD + B) compared to SD group, largely due to a significant reduction in evenness (Simpson’s evenness) in these groups compared to the SD group (Fig. 4A). Significant differences in beta diversity using a global PERMANOVA test (*p* = 0.001) as well as pairwise comparisons (q = 0.0024–0.003) were observed between all treatments using generalized UniFrac distances (Fig. 4B) indicating distinct microbial compositions in different treatment groups by the end of the treatment period.

Individual gut microbiota taxonomic groups were affected by diet and/or the addition of *B. uniformis* CECT 7771 (Fig. 4C). As expected, substantial increases in the genus *Bacteroides* were observed in both mouse groups that were fed *B. uniformis* CECT 7771 (SD + B and HFHFD + B groups) compared to their respective groups (SD and HFHFD), but differences were statistically significant only in obese mice (Fig. 4C). Interestingly, significant increases in the potentially pathogenic genus *Helicobacter* were observed in obese mice under the HFHFD, whereas abundance of this genus was reduced in the HFHFD + B group, which were similar to the control group (SD). Further analysis via BLAST of the DNA sequence associated with the OTU classified to this genus revealed a single species identified as *Helicobacter ganmani* (100% identity). *Ruminococcaceae* UCG-014 was reduced by the HFHFD but the administration of the bacteroides strain did not restore this alteration.

### Correlations between metabolic, immune and gut microbiota features

**Figure 5:**
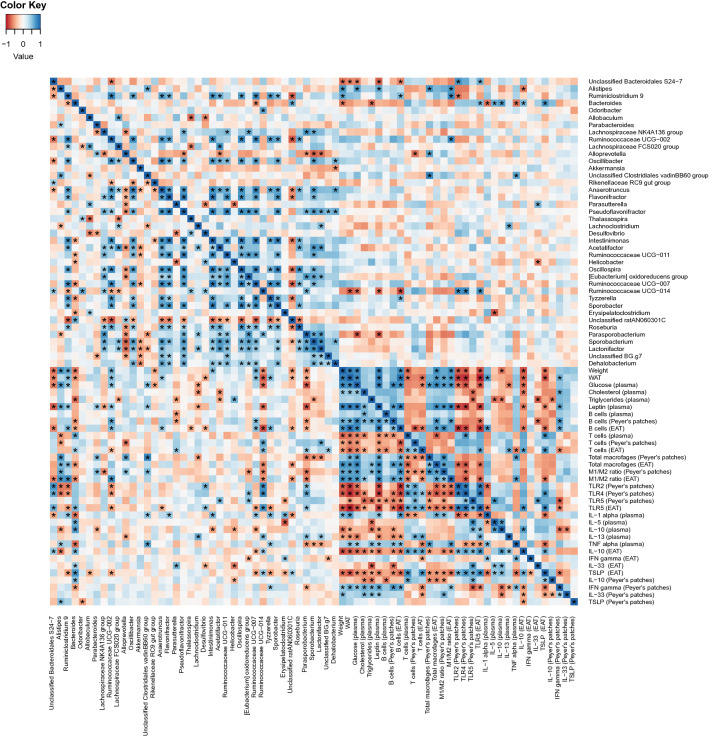
Heatplot of correlations between anthropometric, metabolic, and immune features with the top 50 most abundant gut microbiota taxonomic groups (classified to the lowest taxonomic level). The relative key color indicates the value of Spearman’s correlation coefficient rho (ρ) (blue = positive; red = negative). *p* values were adjusted with the false discovery rate method for multiple comparisons. Variable pairs that have an “*” below the diagonal line are significantly correlated and pairs with a “*” above the diagonal line are significantly correlated after adjustment of *p* values for multiple comparisons (*q* < 0.05).

Increased weight gain was positively correlated (*q* < 0.05) with EAT weight (WAT), blood glucose, leptin, B cells (from EAT), and total macrophages and ratios of M1/M2 (from EAT) (Fig. 5). Negative correlations (*q* < 0.05) were observed between expression of TLR5 in either PP or EAT with obesity markers such as increased body weight gain, cholesterol, triglycerides, EAT weight, blood glucose and leptin as well as the blood pro-inflammatory markers IFNγ and IL-1α, while TLR5 from EAT positively correlated with anti-inflammatory makers IL-10 and TSLP from EAT (Fig. 5). Furthermore, negative correlations (*q* < 0.05) between TLR2 or TLR4 from PP were observed with weight gain, EAT weight, blood glucose and leptin, as well as with B cells and total macrophages (from EAT) and (Fig. 5).

Significant negative correlations (*q* < 0.05) were observed between the genus *Bacteroides* and body weight gain, plasma triglycerides and several blood pro-inflammatory markers (i.e. IL-1α, ΤNFα), while positive correlations were observed for both anti-inflammatory makers in the obesity context (e.g. IL-10 in blood and EAT, plasma IL-5, TSLP from EAT and TLR5 expression in EAT; Fig. 5). Multiple bacterial taxonomic groups were also positively correlated (*q* < 0.05) with TLR5 from EAT (Bacteroidales *S24-7, Bacteroides* and *Ruminococcaceae* UCG-014).

## Discussion

This study shows that a Western-style diet locally impacts the intestine, altering the microbiome´s symbiotic configuration and enhancing the inflammatory tone, two interrelated effects linked to obesity and immune-metabolic dysfunction affecting distant organs. The present pre-clinical study also proves that interventions primarily targeting the intestine can be used to reverse the immune-metabolic deregulation caused by the diet via the cross-talk between the gut and the peripheral tissues affected in obesity, such as the adipose tissue. Specifically, the oral administration of the strain *B. uniformis* CECT 7771 partly re-establishes the state of symbiosis and resets adverse intestinal inflammation, ameliorating the systemic immune-metabolic deregulation induced by the Western diet.

Our study specifically shows that the administration of *B. uniformis* CECT 7771 restored the B and T cell deregulation that is characteristic of diet-induced obesity^25–27^, reducing B cells and increasing Tregs in all body compartments studied. It also reversed the obesity-induced increase in total macrophages and the M1/M2 ratio in intestinal PP and EAT. All of these changes occurred in parallel to the restoration of the metabolic homeostasis in obese mice fed *B. uniformis* CECT 7771. In a previous study, it was specifically proven that diet-induced microbiota changes impact the intestinal adaptive immune system, leading to imbalances in effector T and regulatory cells, and that this was sufficient to trigger metabolic disease in experimental models^28^. Considering also that positive relationships have been established between increases in B cells and parallel reductions in Tregs in obesity models^29^, the production of Tregs seems to be a key cellular mechanism whereby *B. uniformis* CECT 7771 restores the immune-metabolic homeostasis. This is also reflected in the shifts of the key pro-inflammatory (TNFα, IFNγ) and anti-inflammatory (IL-10) cytokines produced by macrophages (M1) and Tregs, respectively^29–31^.

It is noteworthy that *B. uniformis* CECT 7771 attenuated not only intestinal inflammation but also systemic and adipose tissue inflammation, inducing changes in the same regulatory and anti-inflammatory cells (increased Tregs and decreased M1/M2 ratio) and key cytokines (TNFα and L-10). In the adipose tissue, we also observed that the intervention with this bacterium increased the concentrations of intestinal cytokines involved in the expansion of Tregs. In particular, *B. uniformis* CECT 7771 stimulates the production of TSLP, a cytokine known to be regulated by intestinal bacteria and essential for promoting the expansion of Tregs^32,33^. In addition, *B. uniformis* CECT 7771 increases intestinal IL-33 concentrations in the EAT, which is a cytokine reported to promote Treg function. IL-33 signalling in T cells stimulates Treg responses by enhancing transforming growth factor (TGF)-β1-mediated differentiation of Treg cells and providing a necessary signal for Treg-cell accumulation and maintenance in inflamed tissues^34^. Furthermore, IL-33 contributes to orchestrating innate immune cell responses mediated by type 2 innate lymphoid cells (ILC2). In particular, IL-33-mediated ILC2 activation leads to tissue accumulation of eosinophils and M2 macrophages^35^, which is consistent the reductions of the M1/M2 ratio found in the EAT of obese mice fed bacteroides. *B. uniformis* CECT 7771 may also directly stimulate Treg differentiation via similar immunomodulatory molecules such as polysaccharide A, as observed in other *Bacteroides* spp. such as *B. fragilis*^36^, which was shown to depend on TLR2 signalling.

To investigate deeper into the possible molecular mechanisms that could initiate and mediate the effects of *B. uniformis* CECT 7771 on obesity-associated inflammation, we analyzed the expression of TLRs in the intestinal PP and EAT. The Western diet generally reduced expression of TLRs (TLR2, 4, 5) especially in the ileum PP. We speculate that the reduced TLR2/4 expression may be a compensatory mechanism to counteract the inflammatory tone caused by the diet and the associated dysbiosis in obese mice. Indeed, persistent exposure to LPS may cause endotoxin tolerance associated with down regulation of TLR4 expression, which could explain the decreased TLR4 levels in obese human subjects^37^. By contrast, other studies reported increase TLR2 and TLR4 gene expression especially in the adipose tissue of diet-induced obesity in mice. Moreover, the use of knock-out mice or loss of function mutations in either TLR2, TLR4 or MyD88 also confirmed their role in obesity-associated inflammation^38^. Therefore, a better understanding of the role TLR2/4 expression plays, specifically in intestinal PP, in obesity requires further investigations.

In our study, the Western diet exerted the most remarkable effect on TLR5, reducing its expression in both the PP and the EAT. Notably, the expression of TLR5 was completely normalized by the administration of *B. uniformis* CECT 7771 to obese mice in PP and partially normalized in the EAT, but this was not the case for TLR2 and TRL4. Previous studies in TLR5-deficient mice indicated that signaling via this innate immune receptor plays a key role in metabolism since these knock-outs develop features of metabolic syndrome such as hyperlipidemia, insulin resistance, and weight gain, which were also correlated with changes in the gut microbiota^39^. Although strains of *B. uniformis* are not described as being motile and having flagella^40^, we searched for flagellin encoding genes in the whole genome of *B. uniformis* CECT 7771^41^ since TLR5 is known to be activated by bacterial flagellin. However, we could not identify any genes related to flagellin production in this species, indicating that some other ligands from this bacterium may be initiating the activation of TLR5. To confirm whether *B. uniformis *per se or the diet-induced microbiota-changes could be responsible for the effects of the interventions on TLR5 expression in vivo, we conducted experiments using a cell line expressing TLR5. This study showed that the Western diet-induced fecal microbiota changes and/or their metabolites were responsible for the shifts in TLR5 in vivo in obese mice. *Bacteroides*-induced microbiota changes could have led to the production of butyrate and this, in turn, could explain a subsequent increase of TLR5 expression as reported elsewhere^42^. The effects of *B. uniformis* CECT 7771 on TLR expression could also have been a secondary consequence of its effects on leptin levels as suggested in previous studies relating reduced concentrations of leptin with increased expression of TLRs^43^. In addition, our experiments demonstrated that pure cultures of *B. uniformis* CECT 7771 could be directly responsible for TLR5 activation, although the responsible motif eliciting this effect remains unknown.

Experimental studies using flagellin as a ligand of TLR5 suggest that this signaling pathway can contribute to TSLP production, which plays a major role in Th2 polarization of the immune response mediated by myeloid DCs leading to IL-10 production^44^. Evidence from in vitro and ex vivo culture studies also indicate that IL-33 production can be stimulated via TLR5 signalling^45^. Therefore, the activation of TLR5 directly by cellular components of *B. uniformis* CECT 7771 and the microbiota derived metabolites in EAT of obese mice could explain the molecular mechanism by which the administration of this strain increases both TSLP and IL-33 production with downstream effects on Tregs. The increase of Tregs in obese mice fed *B. uniformis* CECT 7771 could also reduce the activation of T effector cells and, thereby, reduce the recruitment and activation of pro-inflammatory macrophages (M1) and the release of innate immune mediators that cause intestinal barrier dysfunction and subsequently enhance WAT inflammation. In our study, the Western-diet induced increases in *Helicobacter* spp., which are known to cause inflammation in murine models^46–48^, could have contributed to elevating the intestinal inflammatory tone of obese mice. In fact, *Helicobacter ganmani* has been demonstrated to increase the expression of the pro-inflammatory cytokine IL12/23p40 in IL10-deficient mice^49^. The ability of *B. uniformis* CECT 7771 to partly restore the intestinal ecosystem reducing the abundance of *Helicobacter* spp., could also have contributed to limiting the expansion of the inflammatory cascade towards peripheral tissues. Consistent with this hypothesis, study models suggest that metabolic inflammation associated with Western diets originates in the intestine before affecting the WAT. The intestine is the first tissue exposed to the diet and also the first to respond by recruiting pro-inflammatory macrophages that, in turn, activate cytokine production and alter gut permeability, ultimately resulting in inflammation and insulin resistance in WAT, while inhibition of intestinal macrophage recruitment prevents insulin resistance^9^. Specifically, using a model of adipose tissue inflammation independent of the diet, it was proven that the microbiota drives metabolic inflammation, affecting ultimately the WAT^50^. Further studies using knock-outs for the monocyte chemoattractant protein CCL2 indicated that gut microbiota is responsible for induction of CCL2, which in turn enhances macrophage accumulation in WAT. The study established gut microbiota as a factor aggravating inflammation during diet-induced obesity and, therefore, as a suitable target for therapies against associated metabolic perturbations^13^, as shown in our study.

All in all, this study reinforces the idea that diet-induced microbiota changes cooperate with obesogenic diets, aggravating the immune-metabolic deregulation in obesity. The findings also suggest that dietary interventions targeting intestinal inflammation can contribute to ameliorating systemic immune-metabolic dysfunction. The identification of molecular targets (TLR5) and mediators (TSLP, IL33 and Tregs) responsible for the immune regulatory effects of *B. uniformis* CECT 7771 in diet-induced obesity also provides new insights into the mechanism whereby effector human bacterial strains can work to attenuate the adverse impact of obesity in metabolic health.

## Materials and methods

### Bacterial strain and culture conditions

*Bacteroides uniformis* CECT 7771 was originally isolated from stools of breast-fed infants, identified by 16S rRNA gene and whole genome sequencing as described previously^41^, and deposited in the Spanish Culture Collection (CECT). The bacteria were grown in Schaedler broth without hemin (Scharlau, Barcelona, Spain) at 37 °C under anaerobic conditions (AnaeroGen, Oxoid, Basingstoke, UK). Cells were harvested by centrifugation (6,000 g for 15 min, at 4 °C), washed twice in phosphate buffered saline (PBS, 130 mM sodium chloride, 10 mM sodium phosphate, pH 7.4), and then re-suspended in 10% skimmed milk. Aliquots of these suspensions were frozen in liquid nitrogen and stored at −80 °C until use for animal trials. After freezing and thawing, the number of live cells was determined by colony-forming unit (CFU) counting on Schaedler agar medium after 48 h incubation. One fresh aliquot was thawed for every new experiment to avoid variability in bacterial viability.

### Experimental design, animals, and diets

The following experimental design was based on previous methods described in Moya-Pérez^12^ and Gauffin Cano^24^. C57BL/6 adult (6–8 weeks) male mice were purchased from Charles River Laboratories (L'Arbresle Cedex, France). In the adaptation period (7 days), animals of each experimental group were housed together in a stainless-steel cage in a temperature-controlled (23 °C) room with a 12-h light/dark cycle and 40–50% relative humidity and were fed a standard diet (SD) ad libitum. Then, mice were randomly divided into four groups (n = 10 mice per group) as follows: (1) SD group, receiving a SD plus placebo (10% skimmed milk); (2) HFHFD group, receiving a high-fat diet supplemented with fructose 20% (HFDFD) plus placebo; (3) SD + B group, receiving SD and a daily dose of 1 × 10^8^ CFU *B. uniformis* CECT 7771 (10% skimmed milk); and (4) HFHFD + B group, receiving HFHFD and a daily dose of 1 × 10^8^ CFU CECT 7771 by oral gavage. To induce obesity, mouse groups 2 and 4 were switched from the SD (lard/corn oil 13% Kcal) administered during the adaptation period to a HFHFD (palm oil 48% kcal) plus fructose (D (-)-Fructose ≥ 99%, Sigma, Saint Louis, USA) in the drinking water and this dietary regime was maintained for 14 weeks. Diet information is detailed in Supplementary Table 1. The HFHFD (S9667-E010 SSNIFF) provided 18% kcal as protein, 34% kcal as carbohydrate and 48% kcal as fat (4.7 kcal/g), whereas the SD (S9667-E020 SSNIFF) provided 23% kcal as protein, 64% kcal as carbohydrate and 13% kcal as fat (3.6 kcal/g), both diets were obtained from Ssniff (Soest, Germany). Mice had free access to water and feed. All experimental procedures were performed in accordance with European Union 2010/63/UE and Spanish RD53/2013 guidelines and approved by the local ethics committee (Animal Production Section, Central Service of Support to Research [SCSIE], University of Valencia, Spain) and authorized by Dirección General de Agricultura, Ganadería y Pesca (Generalitad Valenciana; approval ID 2017/VSC/PEA/00,125). The study was also carried out in compliance with the ARRIVE guidelines (https://arriveguidelines.org). Body weight was measured once a week and stool samples were collected at the end of the experiment. After 14 weeks of dietary intervention, animals were fasted for 16 h, anaesthetized with isoflurane and sacrificed by cervical dislocation. Blood samples were collected in EDTA-containing tubes (two for each animal): one of them was centrifuged (2,000 × g for 10 min at room temperature) and the supernatant (plasma) was kept at −80 °C for endocrine and metabolic marker analysis and the other tube was used for immune cell flow cytometry analysis in plasma. The EAT and the last portion of the ileum containing Peyer's patches were suspended in phosphate buffered saline solution (PBS, 130 mM sodium chloride and 10 mM sodium phosphate, pH 7.4) and kept at 4 °C until further processing for flow cytometry analysis.

### Quantification of endocrine and metabolic parameters

Plasma leptin concentration was determined by the Assay Max Mouse Leptin ELISA kit (ASSAYPRO, Missouri, USA) with a sensitivity threshold of 0.3 ng/mL. Insulin was measured using a Rat/Mouse ELISA kit (Sigma, Sant Louis, USA) with a sensitivity threshold of 0.3 ng/mL. Cholesterol (Cholesterol Liquid kit) and triglycerides kits (Triglyceride Liquid kit) were purchased from Química Analítica Aplicada SA (Tarragona, Spain), and measured according to the manufacturer’s instructions.

### Glucose tolerance test (GTT)

The GTT was performed in vivo after 10 weeks of dietary intervention according to similar methods described in Moya-Pérez^12^. The GTT was performed after 6 h of food deprivation, after which 2.0 g/kg body weight glucose was administered by oral gavage. Blood samples were taken by saphenous vein puncture at baseline and 15, 30, 45, 60, and 120 min after oral glucose administration. Plasma glucose levels were analyzed with glucose test strips (Ascensia Esyfill, Bayer, NY, USA) and a glucometer (Ascensia VIGOR, Bayer, NY, USA), with a detection level ranging from 30 to 550 mg glucose/dL. The area under the glucose curve (AUC) was estimated by plotting the glucose concentration (mg/dL) versus time (min).

### Cytokine quantification

Tissues (Ileum and epididymal adipose tissue [EAT]) were weighed and incubated for 10 min in RIPA buffer (1 × solution, 150 mM NaCl, 1.0% IGEPAL CA-630, 0.5% sodium deoxycholate, 0.1% SDS, and 50 mM Tris, pH 8.0) (Sigma, Madrid, Spain). Samples were then homogenised with a Tissue Ruptor (Qiagen, Madrid, Spain) at 4 °C for 1 min and centrifuged at 10,000 rpm, at 4 °C for 5 min. This method enables efficient cell lysis and protein solubilisation while avoiding protein degradation and interference with the proteins’ immunoreactivity. Supernatants were stored at −80 °C until analyzed.

For cytokine quantification, the Mouse FlowCytomix Multiplex Kits (eBioscience, Affymetrix Company, Vienna, Austria) were used. The following cytokines were analyzed in plasma: IL-1α, IL-5, IL-10, IL-13, and TNF-α (eBioscience, Affymetrix Company, Vienna, Austria) by flow cytometry using a FACS Canto cytometer (Becton Dickinson, NJ, USA). Sensitivity thresholds for each cytokine were: IL-1α: 15.7 pg / mL, IL-5: 4.0 pg / mL, IL-10: 5.4 pg / mL, IL-13: 9.3 pg / mL and TNF-α: 2.1 pg / mL. Data are expressed as pg cytokine/mL of plasma. In addition, IL-10, IFN-γ, IL-33, and thymic stromal lymphopoietin (TSLP) were determined in ileum and epididymal WAT using ELISA kits (Biolegend, San Diego, CA and eBioscience, Affymetrix, San Diego, CA for TSLP). Sensitivity thresholds for each cytokine were: 16 pg/mL for IL-10, 4 pg/mL for IFN-γ, 25 pg/mL for IL-33 and 16 pg/mL for TSLP.

### Quantification of lymphoid and myeloid cells and TLR expression by flow cytometry

Peripheral blood, EAT, and Peyer's patches (PP) from small intestine (ileum) were used for immune-cell analysis by flow cytometry. For adipose tissue, visible vessels and connective tissue were carefully removed and the tissue was minced with fine scissors and digested with 0.15% collagenase type II from *Clostridium histolyticum* C6885 (Sigma, Saint Louis, USA) in FACS buffer (PBS with 0.5% BSA and 2 mM EDTA) at 37 °C for 30 min. The PP were washed with FACS buffer, cut into small pieces and incubated with collagenase type I from *Clostridium histolyticum* C9891 (Sigma, Saint Louis, USA) at 37 °C for 30 min. Afterwards, the digested tissues were passed through 40 μm mesh filters and washed in FACS buffer, then centrifuged at 2,000 rpm for 5 min at 4ºC and the pelleted cells were stained and analyzed. To analyze immune markers in peripheral blood, 100 μL were resuspended in antibody solution for 30 min in darkness, then mixed vigorously with 2 mL FACS buffer (160 mM NH_4_Cl, 0.1 mM EDTA, 12 mM NaHCO_3_) to lyse red blood cells for 10 min at room temperature. The samples were centrifuged at 2,000 rpm for 5 min, the pellet was washed twice with 2 mL FACS buffer, resuspended in 300 μL FACS buffer and analyzed by flow cytometry.

Cells were stained with the following fluorescent dye-labelled mouse monoclonal antibodies for lymphoid cell analysis: CD3^FITC^, CD4^BV510^, CD8^APC^, CD25^PE^, and CD19^BV421^. The following cellular subsets were analyzed for myeloid cell analysis: total lymphocytes (CD3 +), regulatory T cells (CD3 + CD4 + CD25 +), and B cells (CD3-CD19 +). The following cellular subsets were analyzed: total macrophages (F4/80 +), M1 macrophages (F4/80 + CD11c + CD206-) and M2 macrophages (F4/80 + CD11c-CD206 +). In addition, CD282^FITC^ and CD284^PE^ antibodies were used to determine TLR2 and TLR4, respectively, in PP. For detection of TLR5, primary (rabbit anti-mouse TLR5) and secondary (goat anti-rabbit IgG^PerCP-Cy5.5^) antibodies were used in PP and EAT. All conjugated antibodies were from BD Biosciences (San Jose, CA, USA) except for CD206 and TLR5 that were from BioLegend (Fell, Germany) and from Santa Cruz (Heidelberg, Germany), respectively. All antibodies were used according to the manufacturer’s instructions. After washing, cells were analyzed with BD LSRFortessa and BD FACSVerse cytometers (Becton Dickinson, NJ, USA). The data were analyzed using BD FACS DIVA Software v.7.0. and BD FACS Suite Software v.1.0.3.2942.

### TLR5 activation assays in human HEK-Blue h TLR5 cell cultures

To test whether pure cultures of *B. uniformis* CECT 7771 as well as fecal samples from mice exposed to the different experimental treatment conditions stimulate TLR5, in vitro experiments using a HEK293 cell line were carried out. The HEK293 cell line stably transfected with human TLR5 (HEK-Blue hTLR5 cells) were obtained directly from Invivogen (CA, USA). TLR5 activity can be determined by measuring embryonic alkaline phosphatase (SEAP) in which production is induced by NF-κB and AP-1 after TLR5 activation. Levels of SEAP can be determined with HEK-Blue Detection (Invivogen), a cell culture medium that allows for real-time detection of SEAP. HEK-Blue hTLR5 cells were grown and cultured up to 70–80% confluency using as a maintenance medium Dulbecco’s Modified Eagle Medium (DMEM) supplemented with 4.5 g/l D-glucose, 10% fetal bovine serum (FBS), 50 U/ml penicillin, 50 μg/ml streptomycin, 100 μg/ml Normocin and 2 mM L-glutamine. Cells were seeded into flat-bottom 96-well plates and resuspended in HEK-Blue Detection (25,000 cells/well). The 96-well plates were incubated for 6 h at 37 °C in a 5% CO_2_ incubator. Fecal samples from the different mouse groups (SD, HFHFD and HFHFD + B) and pure cultures of *B. uniformis* CECT 7771 were used as different stimuli. Fecal samples were previously diluted in 1X PBS buffer (1:10 w/v final) and submitted to low speed centrifugation (2,000 × g for 10 min at 4 °C) to eliminate particulate material (20 μl). Cell suspensions of *B. uniformis* CECT 7771 were adjusted to final concentrations of 1:100 HEK293 cells:bacterial cells. Recombinant flagellin (RecFLA-ST, 1 μg/ml) and cell suspensions of pure cultures of *Clostridium butyricum* were used as a positive control while endotoxin-free water and cell suspensions of pure cultures of a strain of *Phascolarctobacterium faecium,* a known species lacking flagellin*,* were used as negative controls. SEAP secretion was detected after 16 h of stimulation by measuring the OD_600_ in HEK-Blue h TLR5 supernatant using a Spectrophotometer (Multiskan Spectrum, Thermo Fisher Scientific).

### Analysis of gut microbiota

Processing of samples for gut microbiota analysis was carried out according to the methods described in González-Ramos^51^. Fecal samples from individual mice from each experimental group were collected at the end of the intervention and were immediately frozen in liquid nitrogen and stored at −80 °C until processing. DNA extraction was carried out using a Fast DNA Stool Mini Kit (Qiagen) according to the manufacturer’s instructions with several modifications. First, fecal samples (up to 220 mg) were added to sterile 2 mL tubes filled with glass beads and one ml of Inhibitex buffer (Qiagen) was added to each tube. Samples were homogenized using a beadbeater for 2 successive rounds for 1 min and then heated to 95 °C for 10 min. Samples were amplified in triplicate via PCR using primers (S-D-Bact-0563-a-S-15 / S-D-Bact-0907-b-A-20) that target the V4-V5 variable regions of the 16S rRNA gene^52^. Samples were tagged with barcodes to allow multiplexing during the sequencing process. Triplicate reactions consisted of final concentrations of Buffer HF (1X), dNTPs (0.11 µM) primers (0.29 µM each) and Taq Phusion High Fidelity (0.007 U/µL) in final volumes of 35 µL. Cycling conditions consisted of 98 °C for 3 min, followed by 25 cycles of 95 °C for 20 s, 55 °C for 20 s, and 72 °C for 20 s, followed by a final extension step of 72 °C for 5 min. Triplicate sample amplicons were combined and purified using the Illustra GFX PCR DNA and Gel Band Purification Kit (GE Healthcare) according to the manufacturer’s instructions and combined in equimolar concentrations before carrying out sequencing on a MiSeq instrument (Illumina). All raw sequence data has been submitted to ENA-EMBL Accession number: (PRJEB22917).

Bioinformatic processing of data was carried out using the software QIIME^53^, Mothur^54^, and UPARSE^55^. Briefly, using QIIME, paired-end forward and reverse Illumina reads were joined into contigs, barcodes were extracted and reads were demultiplexed. Primers were then removed using the software program Mothur. Using UPARSE, chimeras were removed and reads were clustered at 97% identity into OTUs using default settings. An OTU abundance table was generated within the UPARSE pipeline by mapping reads to representative sequences for each OTU. Using QIIME, a biom file was created from the OTU table and reads were rarefied and singletons were removed. A phylogenetic tree was constructed from representative sequences for each OTU, and aligned using PYNAST^56^ and filtered using default settings. Alpha diversity metrics (Shannon’s, Simpson’s, and Simpson’s reciprocal diversity index, Simpson’s evenness) were calculated. Beta diversity analysis was conducted using generalized UniFrac (GUniFrac)^57^ and principal coordinates analyses (PCoA). Samples were classified taxonomically with Mothur using taxonomic assignments and full-length sequences from the SILVA database (release 123)^58^.

### Statistical analyses

Data from animal experiments were analyzed using Graph Pad Prism software (LaJolla, CA). Data distribution was assessed by the Kolmogorov–Smirnov normality test. For normally distributed data, differences were determined with one or two-way ANOVAs (as appropriate) *and *post hoc Bonferroni’s tests. Non-normally distributed data were analyzed with the non-parametric Mann–Whitney *U* test. In every case, *p* values < 0.05 were considered statistically significant. Gut microbiota statistics and data visualization of sequencing data were carried out using the R statistical software and related R packages or QIIME^53,59^. Comparison between dietary groups of relative abundances of taxonomic groups was carried out using a Kruskal–Wallis test followed by a Wilcoxon rank-sum test to identify significant differences. All *p* values were corrected for multiple comparisons using false discovery rate where (*q* < 0.05) was a cutoff for significance. Comparisons of beta diversity between dietary groups using generalized UniFrac distances were performed by generating a principle coordinates analysis (PCoA) and conducting PERMANOVAs with adonis() within the GUniFrac package in R. Correlations between gut microbiota taxonomic groups and biochemical and immunological parameters were performed using Spearman’s rank correlation coefficients (ρ) using the (cor function) and *p* values were adjusted with the false discovery rate method for multiple correlations. Correlation plots were visualized using the R heatmap.2() function.

## Supplementary Information

Supplementary Information.
