# Supplementary material for: Bacteroides uniformis CECT 7771 alleviates inflammation within the gut-adipose tissue axis involving TLR5 signaling in obese mice
Source: Sci Rep. 2021 Jun 3;11:11788. doi: 10.1038/s41598-021-90888-y (PMC8175583; doi:10.1038/s41598-021-90888-y)
Supplement: Supplementary file 1 — Supplementary Information. [file 41598_2021_90888_MOESM1_ESM.docx]

**Supplementary Table 1. Composition of standard diet (SD) and high-fat-high fructose diet (HFHFD)**

|  | | **SD**  **S9667-E020 S5745-E707 5** **%** **fat** **(13** **kJ%)** | **HFHFD**  **S9667-E010 S5745-E712 25** **%** **fat** **(48** **kJ%)** |
| --- | --- | --- | --- |
| Casein | % | 24.00 | 24.00 |
| Corn starch | % | 47.80 | 27.80 |
| Maltodextrin 10 DE | % | 5.60 | 5.60 |
| Sucrose | % | 5.00 | 5.00 |
| Cellulose | % | 5.00 | 5.00 |
| L-Cystine | % | 0.20 | 0.20 |
| Vitamin premix | % | 1.20 | 1.20 |
| Mineral & trace element premix | % | 6.00 | 6.00 |
| Choline Cl | % | 0.20 | 0.20 |
| Pork Lard | % | 1.50 | —— |
| Corn oil | % | 3.50 | —— |
| Palm oil | % | —— | 20.00 |
| Soybean oil | % | —— | 5.00 |
| Protein | % | 21.10 | 21.10 |
| Fat | % | 5.10 | 25.10 |
| Fiber | % | 5.00 | 5.00 |
| Ash | % | 5.40 | 5.40 |
| NfE (carbohydrates) | % | 58.80 | 39.60 |
| ME (Atwater) | MJ/kg | 15.30 | 19.70 |
|  | **kcal/kg** | **3655** | **4706** |
| Protein | kcal% | 23 | 18 |
| Fat | kcal% | 13 | 48 |
| Carbohydrates | kcal% | 64 | 34 |
| ***Fatty*** ***acids*** *,* *%* *in* *the* *diet* |  |  |  |
| C12:0 |  | 0.01 | 0.01 |
| C14:0 |  | 0.04 | 0.21 |
| C16:0 |  | 0.78 | 9.18 |
| C18:0 |  | 0.28 | 1.11 |
| C20:0 |  | 0.02 | 0.10 |
| C16:1 |  | 0.05 | 0.05 |
| C18:1 |  | 1.56 | 9.19 |
| C18:2 |  | 2.08 | 4.67 |
| C18:3 |  | 0.05 | 0.35 |
| C20:4 |  | 0.03 | —— |
